# The Potential Neuroprotective Role of Free and Encapsulated Quercetin Mediated by miRNA against Neurological Diseases

**DOI:** 10.3390/nu13041318

**Published:** 2021-04-16

**Authors:** Tarek Benameur, Raffaella Soleti, Chiara Porro

**Affiliations:** 1 College of Medicine, Department of Biomedical Sciences, King Faisal University, Al-Ahsa 31982, Saudi Arabia; tbenameur@kfu.edu.sa; 2 Univ Angers, Université de Nantes, Inserm, CRCINA, SFR ICAT, F-49800 Angers, France; raffaella.soleti@univ-angers.fr; 3 Department of Clinical and Experimental Medicine, University of Foggia, 71121 Foggia, Italy

**Keywords:** quercetin, natural flavonoid, neuroinflammation, microglial cells, miRNA, neuroprotection, neurodegenerative diseases, antioxidant, nanodrug

## Abstract

Chronic neuroinflammation is a pathological condition of numerous central nervous system (CNS) diseases such as Parkinson’s disease, Alzheimer’s disease, multiple sclerosis, amyotrophic lateral sclerosis and many others. Neuroinflammation is characterized by the microglia activation and concomitant production of pro-inflammatory cytokines leading to an increasing neuronal cell death. The decreased neuroinflammation could be obtained by using natural compounds, including flavonoids known to modulate the inflammatory responses. Among flavonoids, quercetin possess multiple pharmacological applications including anti-inflammatory, antitumoral, antiapoptotic and anti-thrombotic activities, widely demonstrated in both in vitro and in vivo studies. In this review, we describe the recent findings about the neuroprotective action of quercetin by acting with different mechanisms on the microglial cells of CNS. The ability of quercetin to influence microRNA expression represents an interesting skill in the regulation of inflammation, differentiation, proliferation, apoptosis and immune responses. Moreover, in order to enhance quercetin bioavailability and capacity to target the brain, we discuss an innovative drug delivery system. In summary, this review highlighted an important application of quercetin in the modulation of neuroinflammation and prevention of neurological disorders.

## 1. Introduction

The search for novel natural therapeutic agents to prevent or slow the progression of neurodegenerative diseases is gaining great attention. Indeed, recent evidence has affirmed that the consumption of fruits and vegetables is strictly linked to a decreased risk of developing a large variety of age-related and neurodegenerative diseases.

Quercetin is a natural bioflavonoid found abundantly in fruit and vegetables such as apples, berries, onions and capers. It was firstly isolated by the physiologist Albert Szent- gyorgyi de Nagyrapolt, a Nobel Prize recipient for Physiology/Medicine in 1936 [1].

Quercetin has multiple pharmacological properties, including neuroprotective, anti- cancer, cardioprotective, antioxidant, antiviral, antimicrobial, antithrombotic, antiapoptotic, anti-inflammatory and hepatoprotective. It is also used as potential treatment for severe inflammation [2].

The glial cells of the central nervous system (CNS) consist of oligodendrocytes, astrocytes, microglia and ependymal glial cells. However, the peripheral nervous system (PNS) is composed of other glial cells such as Schwann cells, and satellite glial cells, which provide nutrients and structural support to neurons. Glial cells, an essential part of the CNS and immune system, provide homeostatic support, protection, defense against pathogens, and neuronal maintenance. Upon activation by tissue injury and inflammation, they release inflammatory mediators and induce neuroinflammatory diseases. Also, the blood–brain barrier (BBB) is maintained by the homeostasis of CNS microenvironment, and acts as a physical and metabolic barrier for limiting the movement of substances into the brain.

Quercetin is known for its multiple proven health benefits including its protective role in neurodegenerative diseases due to its capacity, among other, to modulate microRNA (miRNA) expression. miRNAs have been shown to regulate critical processes, including inflammation, differentiation, proliferation, apoptosis and immune responses as well as neurodegeneration. To improve the bioavailability and stability of quercetin many researchers have developed nano-formulations to increase bioavailability of quercetin.

The current review article collects the latest studies on protective effects of quercetin in brain cells, with particular focus on microglia cells.

We also elaborate on the regulation of miRNA expression by quercetin in neurodegenerative diseases and on the recent advancements for new formulation of quercetin for increase its effectiveness.

## 2. Quercetin and Its Dietary Sources

“Quercetin” is derived from the Latin word “quercetum,” and literally means “oak forest”. Quercetin is a natural and bioactive flavonoid which is not produced in the human body [3,4]. Most common forms of quercetin described in the literature are quercetin glycoside, quercetin sulfate, quercetin glucuronide and methylated quercetin [5].

Quercetin is the most abundant flavonoid in vegetables and fruits such as onions, broccoli, berries, grapefruit, apple, black and green tea, red grapes, citrus fruits, green leafy vegetables and beans [6,7]. The required human dietary intake of all flavonoids is estimated between 200 and 350 mg/day, while the normal daily ingestion of flavanols varies from 20 to 35 mg, of which quercetin accounts for nearly 50%, with a daily consumption of about 10 mg/day [8,9]. However, the ingested amount with the diet is subject to many variables essentially the plant composition, dietary habits, age and other factors.

Like other flavanols, after ingestion, the primary site of quercetin absorption is the small intestine and only minor proportion of quercetin is absorbed in the stomach. The conjugated quercetin is transported and modified in the liver before re-entering the systemic circulation and transported to the muscles and brain. Quercetin produced metabolites after undergoing phase I and II metabolism in the liver then transported to the body tissues through the circulating blood [10,11]. The main metabolites found in urine after ingestion of quercetin are quercetin—diglucuronide, -3′-glucuronide, isorhamnetin-glucuronide, glucuronide sulfate, and -methyl quercetin diglucuronide. In total, 23 metabolites were identified, with twelve being quantified in the urine and only five in plasma [12]. However, quercetin-3-O-β-D-glucuronide, is the most abundant metabolite of quercetin, which contributes to the activation of many physiological functions with beneficial effects when distributed in the tissues [13]. 

Quercetin has been proven to possess various biological properties including its antioxidant and anti-inflammatory roles in many inflammatory, metabolic and neurodegenerative diseases [5]. This may ameliorate the overall health and contributes to diseases prevention [14]. In addition, quercetin exhibits other physiological properties, including antithrombotic, anti-ischemic, antiapoptotic, antitumoral, antibacterial and antiviral activities [15,16,17].

More interestingly, a growing body of evidence has shown that quercetin has a broad therapeutic potential for the treatment and prevention of various diseases including cancer, cardiovascular and neurodegenerative diseases. Hence, it is considered as neurohormetic phytochemical with potential neuroprotective effects associated with reduced levels of oxidative stress [2]. 

In general, the oral administration of quercetin in humans was well tolerated and safe; adverse effects have been rarely reported [14,18,19]. However, the biosafety assessment of the long-term use of high doses of quercetin in human requires further investigation.

## 3. Broad Mechanisms of Action of Quercetin

Quercetin has benzo-(γ)-pyrone skeletal structure with a C6-C3-C6 carbon framework, which consists of two benzene rings linked through a heterocyclic pyrone ring. Quercetin (3,3′,4′,5,7-pentahydroxyflavone) is known for having five hydroxyl (OH) groups that may undergo glycosylation to quercetin glycosides, which constitutes the major quercetin derivatives.

This modification can change the absorption, solubility, and the in vivo effects of quercetin. It is critical to note that there is a strong relationship between the structural activities of quercetin and its derivatives on the anti-inflammatory and antioxidant activities [20,21].

The protective effects of quercetin are exerted by the chelating activity of divalent cations, scavenging free radicals, and protecting DNA from damage, in addition to their preventive effects against lipid peroxidation and free radical-mediated cytotoxicity [22]. Quercetin not only lowers the serum triglyceride level but also shows protective potential on digestive enzyme activity, antioxidant effect in the hepatopancreas and improve growth performance in freshwater fish (Tilapia) [23,24] and in dietary-induced obese mice [25]. Interestingly, it has been reported that quercetin reduced the triglyceride level via the PPARα cascade in cultured hepatocytes from a chicken, leading to a decreased level of lipid deposition and may reduce the chronic inflammation associated with lipid accumulation [26].

The chemical structure of quercetin guarantees potent antioxidant activity. Indeed, being an excellent scavenger of reactive oxygen species (ROS) and reactive nitrogen species (RNS), quercetin becomes promising candidate to reduce endoplasmic reticulum stress (ER-stress), i.e., an important contributor to inflammation. Furthermore, quercetin attenuated NF–kB activity, a key mediator of inflammation, thereby directly decreasing the cytokine production via this transcription factor. Another study highlighted that quercetin has a profound effect on the activation of the Nuclear factor erythroid 2-related factor 2 (Nrf2) antioxidant signaling pathway and the expression of its associated downstream effector phase II detoxification enzyme glutathione-S-transferase (GST)P1 in skin HaCaT keratinocytes and the human foreskin fibroblasts (BJ cells) [27]. 

Another study by Liu X et al. [28] reported that quercetin had significantly improved cardiac function by reducing myocardial injury and the infarct size. In addition, the in vivo and in vitro quercetin treatment induced a remarkable improvement of myocardium oxidative damage and apoptosis. Quercetin effects involved the reduction of NF–kB activation cascade as a consequence of myocardial ischemia-reperfusion damage [27].

The investigation of the molecular pathways underlying the neuroprotection mechanisms of quercetin in in vitro and in the mouse model of Parkinson’s Disease (PD) has shown that the activation of PKD1–Akt pathway via the upregulation of PPAR-gamma coactivator 1-alpha (PGC-1a) and the transcription factor A, mitochondrial (TFAM) represents a mechanism to restore mitochondrial function and decrease the progression of dopaminergic neurodegeneration [29,30]. Moreover, during endotoxic stress, quercetin increase heme oxygenase (HO-1) expression via mitogen-activated protein kinase (MAPKs). Taken together, this evidence supports the central function of quercetin in regulating inflammation in microglial cells.

Furthermore, quercetin exerts physiological functions on many organs including liver, brain, kidney, blood vessels, muscle, skin, intestine and bone. Mounting evidence suggests that quercetin can influence neurodegenerative diseases, mood disorders, atherosclerosis, and metabolic syndrome [13]. In addition, quercetin treatments of neurodegenerative diseases are able to modulate the expression levels of pro-inflammatory, anti-inflammatory cytokines and chemokines [31].

Studies on cellular, human, and animal models have demonstrated that the strengthened antioxidant, anti-inflammatory, anticancer and neuroprotective activities of quercetin can be achieved through targeting multiple signaling pathways and the downstream effectors gene expression involved in these processes [32]. This includes antiapoptotic effects of quercetin against cervical cancer cells growth [33], anti-oxidative, anti-ER-stress effects associated with diabetic encephalopathy and atherosclerosis [34,35]. Other potential neuroprotective effects of quercetin were shown in various study models such as: diabetes-induced nerve injury [36], mouse model of neurotoxicity [37,38] and CPF-induced neurotoxicity in animal models. 

The modulatory effect of quercetin on inflammatory processes involved a variety of signaling pathways, including its interaction with phosphatidylinositol-3-phosphate kinase (PI3K), MAPK, extracellular signal regulated kinase (ERK), kinase (MEK1), and others [29]. It may also inhibit the activity of PI3K by shifting ATP binding from PI3K and activate AMP-activated protein kinase (AMPK), which triggers antitumoral and anti-inflammatory responses [13]. Inflammation commonly seen in an obesity context is known to induce meta-inflammation described as chronic and low-grade inflammation [39,40,41]. This chronic inflammation induced a release of proinflammatory cytokines and infiltration of macrophages into adipose tissue that is in close association with the progression of insulin resistance via the crosstalk of the insulin-signaling pathway in adipose tissue and skeletal muscle. Importantly, it has been suggested that the quercetin inhibited the synthesis and secretion of proinflammatory mediators and consequently it improves insulin resistance in this context.

Furthermore, another pathway involved in obesity inflammation is those triggered by toll-like receptor 4 (TLR4) [42]. Indeed, TLR4 plays a modulatory role in immune responses, stimulates the release of pro-inflammatory chemokines and cytokines, [43] and the activation of NF–kB signaling pathways [44,45,46]. This deleterious signaling pathway could be modulated by quercetin, which possesses the ability to decrease inflammation by acting on the TLR4/NF–kB pathway [47].

Given the fact that CNS functions are strongly influenced by the gastrointestinal microbiota, it increasingly becoming obvious that the gut microbiota is strongly connected to the CNS through various bidirectional pathways involving immune, endocrine and neuronal pathways. This bidirectional communication can be achieved via the microbiota production of a large number of mediators/substances that can act either locally or at distant and regulating the CNS functions [48]. More interestingly, a study by Xie et al. [49] reported that quercetin treatment is able to influence gut microbiota in diabetic peripheral neuropathy rats by protecting axon and myelin damage from oxidative stress caused by chronic hyperglycemia [49]. Taken together, this suggests that quercetin may exert its indirect neuroprotective role via microbiota involving ROS pathways suppression and other unknown pathways that need further investigation [49].

Quercetin has an interesting inhibitory effect on inflammatory responses: from one side, it inhibits the expression of different components of NLRP3 inflammasome, including the pro-IL-1β; from the other side, it impedes the activation of other signaling pathways, particularly NF–kB [2]. All triggered mechanisms converge to suppress inflammation.

**Figure 1:**
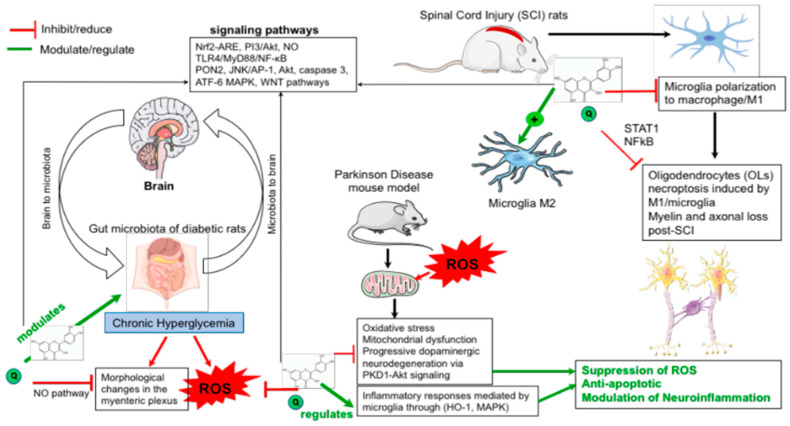
Protective effects of quercetin through modulating microbiota and reactive oxygen species levels to prevent neurological disorders. Quercetin exerts its indirect neuroprotective effect through microbiota involving reactive oxygen species (ROS) pathways suppression in diabetic peripheral neuropathy rats. It regulates cytoprotective protein expression against ROS-induced oxidative stress and suppresses neuroinflammatory responses induced by ROS. Quercetin inhibited inflammatory responses in microglial cells and up-regulate heme oxygenase-1 (HO-1) against endotoxic stress through MAPKs.

In addition to its regulatory roles in energy metabolism and mitochondrial function [50,51], it is widely believed that the Nrf2 cascade exerts antioxidant effects and participates in cell protection and maintaining the redox homeostasis through the subsequent induction of cytoprotective protein expression [22]. Indeed, a great number of genes involved in antioxidant responses, including HO-1, GST, catalase, superoxide dismutase (SOD), sulfidorexin, thioredoxin reductase-1, glutamate cysteine ligase, NADPH, quinone oxidoreductase-1, are under Nrf2 controls [52,53]. Moreover, the interaction of Nrf2 with other signaling molecules modulate the efficiency of the cellular stress response. Beyond its fundamental function in the regulation of the inflammatory responses in various diseases, the activation of Nrf2-ARE has shown neuroprotective effects against neurodegenerative diseases [54]. Figure 1 summarizes the protective effects of quercetin through modulating microbiota and ROS levels for the prevention of neurological disorders.

## 4. Glial Cells and Quercetin-Induced Neuroprotection

The brain is populated by two type of cells: neurons and glial cells. Glial cells, including astrocytes, oligodendrocytes, and microglia, were firstly studied as cells that just support neurons, but now they are considered indispensable constituents for neurons in cellular functions and diversity [55,56]. Glial cells are, in fact, essential players in CNS because they are involved in its development, homeostasis, neuronal communication and contribute to CNS degeneration/regeneration during disease or injury [57]. 

Neuroinflammation is a defense mechanism that initially protects the brain by promoting tissue repair and removing or inhibiting diverse pathogens, but the persistence of inflammatory responses is detrimental and inhibits neuronal regeneration.

The activation of resident glial cells, recruitment and infiltration of peripheral blood cells into the brain parenchyma are among the characteristics of neuroinflammation [58]. During the inflammatory responses many inflammatory mediators, such as cytokines chemokines, increased levels of prostaglandins (PGs), particularly prostaglandin E2 (PGE2) and ROS and RNS. The increasing amount of these species leads to blood–brain barrier (BBB) dysfunction, which in turn results in cellular injury and neuronal dysfunctions [59].

Although neuroinflammation is not it itself an initiating factor in neurodegenerative pathologies, different evidence led to the hypothesis that sustained inflammatory responses involving glial cells activation contribute to neural diseases progression. Neuroinflammation remains a common feature in a number of brain diseases, including Alzheimer’s disease (AD), PD, traumatic brain injury (TBI), amyotrophic lateral sclerosis (ALS), and Huntington’s disease [60].

Microglia represent 5–12% of the total cell population in the mouse brain, are ubiquitously distributed in the brain and are the principal innate immune cells and first responders to pathological insults [61,62]. In a healthy brain, microglia are present in a resting state with ramified morphology containing highly branched processes. After activation, microglia retract their processes, becoming amoeboid in shape. Although different microglia subtypes were previously described under pathological conditions, microglial activation is represented by two main phenotypes, M1 (classical activation) and M2 (alternative activation) [63]. The activated M1 Microglia by lipopolysaccharides (LPS)/interferon gamma (IFN-γ) were able to upregulate pro-inflammatory mediators, including IL-6, IL-1β, ROS, iNOS, and tumor necrosis factor-alpha (TNF-α). These inflammatory mediators will further generate other inflammatory cascade resulting in increased brain injury that later may compromise BBB integrity. M2 microglia is achieved upon stimulation with IL-4, IL-10, IL-13 or TGF-β, and triggers upregulation of anti-inflammatory genes including arginase-1, mannose receptor (CD206), YM1, and FIZZ [64,65]. In CNS, TLR-4 triggered microglia [66,67], are the main source of ROS RNS, TNF-α, and glutamate which are neurotoxic if released at a high dose, as observed in the case of AD, MS, PD, and ALS patients [68,69].

Astrocytes are the most dominant brain glial cells [70]. Recent researchers found that they play active and critical roles in brain homeostasis [71]. Astrocytes regulate blood flow, maintain the BBB, provide energy metabolites to neurons, modulate synaptic activity, control neurotrophin secretion, remove dead cells, and regulate the extracellular balance of ions, fluid and transmitters, and scar formation [70,71,72]. In the course of CNS disease and injury, they are activated, and this process is known as reactive astrogliosis, contributing to both inflammation and reparative processes [73].

Like microglia, astrocytes have been shown to have pro-inflammatory and immunoregulatory or neuroprotective subpopulations. The pro-inflammatory reactive astrocytes upregulate several genes expression including the complement cascade genes and induce the expression of various pro-inflammatory factors such as IL-1β, TNF-α, and nitric oxide (NO) [74]. 

By contrast the anti-inflammatory cytokines, such as IL-4, IL-10 and IL-13 may induce neuroprotective activation of astrocytes, and these alternatively activated astrocytes may release TGF-β, IL-4 and IL-10 [71]. 

Neuroinflammation remains a neuroprotective mechanism, even if several studies suggested that sustained neuroinflammation may induce neurotoxicity and is related to neurodegeneration [75,76].

Exploration of novel natural therapeutic agents in the field of neurodegenerative diseases has gained a remarkable clinical interest in recent past. As reported above, due to its anti-oxidative and anti-inflammatory properties, quercetin consumption has been shown to have potential human health benefits [77]. 

The therapeutic potential of quercetin has been proved in several studies using both in vitro CNS cellular models and in vivo model systems of neuroinflammation.

In glial cells, quercetin acts as anti-inflammatory agent acting with different mechanisms. In rat neuronal cells (PC12 cells) and zebrafish models, quercetin suppressed inflammation caused by 6-hydroxydopamine (6-OHDA) toxicity by decreasing the expression of iNOS and other pro-inflammatory genes [78].

The inhibitory effect on iNOS expression has been confirmed in LPS-stimulated BV-2 microglial cells [79], where quercetin is also able to down regulate the extracellular signal regulated kinase, c-Jun, N-terminal kinase, AKT, Src, Janus kinase-1, activating protein-1 (AP-1) and to enhance the expression of HO-1 [80].

In N9 glial cells, quercetin was able to reduce the LPS-induced mRNA expression of cytokines TNFα and IL-1α [81]. 

Fan et al. reported that quercetin exerts its neuroprotective effect by reducing the effect of microglia polarization. Quercetin treatment, in fact, improves functional recovery after SCI by inhibition of macrophages/microglia polarization to M1 creating a permissive environment that leads to the survival of oligodendrocytes [82].

Yang and coworkers have demonstrated that quercetin reduces obesity-induced hypothalamic inflammation with an inhibition of microglia-mediated inflammatory responses, and this beneficial action is associated with HO-1 induction [83].

In oxygen glucose deprivation (OGD) microglia, quercetin is able to protect cells from damage and reversed the changes induced by stress-related molecules. Quercetin, in fact, suppressed OGD-induced expression of inflammatory genes in BV2 cells and inactivated TLR4/MyD88/NF–kB signaling [84].

The effects of quercetin are not only limited to microglial cells but involve also astrocytes, important cells that generate neuroinflammation inducing neuronal damage by releasing inflammatory and neurotoxic factors, Sharma et al. in fact, found that quercetin decrease the release of IL-6, IL-8, monocyte chemoattractant protein-1 (MCP-1) and ROS production induced by IL-1β [85].

In rat glioma cells (C6 cells) treated with tert-butyl hydroperoxide and H_2_O_2_ quercetin protects cells by reducing ROS production, decreasing apoptotic cells and stimulating HO-1 expression [86]. Also, in rat oligodendrocytes (OLN-93 cells), quercetin increased their survival [87] by modulating NF–kB signaling [88] and inducting paraoxonase 2 (PON2) pathway [89].

In neonatal hypoxic ischemia (HI), quercetin treatment reduces the incidence of the newborn brain damage caused by HI. This effect involves the attenuation of cortical cell apoptosis, as well as suppression of apoptotic marker Bax, and the activation of anti-apoptotic marker Bcl-2. Quercetin decreased the number of cortical cells microgliosis and astrogliosis induced by HI and cortical inflammation. This neuroprotective function on HI in the brain required the inhibition of TLR4/NF–kB signaling pathway [47]. Quercetin has protective effect against the Vincristine-induced apoptosis in the peripheral nerve damage by suppressing essentially Nrf2, Akt and NF–kB pathways in rats [90]. Another study reported that quercetin has anti-apoptotic effects on hippocampus neuronal cells in D-galactose-induced apoptosis in aging mouse models. In this model, quercetin activated the Nrf-2 and its subsequent downstream proteins such as HO-1, SOD [37]. 

Quercetin increases the expression of PON2 in brain cells, both in vitro and in vivo models [22,91]. This neuroprotective potential is significantly reduced in cells lacking PON2, suggesting that PON2 induction by quercetin represents an important target in the prevention of neuronal cells against the oxidative challenges. 

Ghahremani et al. showed that the oxidative damage of the brain induced by chlorpyrifos (CPF) is improved by quercetin treatment, this effect is achieved through the upregulation of PON2 pathway and potentiation of its downstream antioxidant defense mechanisms. The authors suggested that these positive effects of quercetin may be used as a dietary supplement to the CPF-exposed individuals, such as farmers and prevent the CPF-induced neurological disorders [92]. One more positive effect of quercetin in the brain is that it preserves the BBB integrity in the hippocampus of rats treated with polychlorinated biphenyls (PCBs). In this case, quercetin is able to increase tight junction proteins and brain derived neurotropic factor (BDNF) signaling molecules [93]. Moreover, Zhao et al. demonstrated that BBB dysfunction induced by global cerebral I/R injury is attenuated by the quercetin-induced Wnt/β catenin signaling pathway [94].

Recent published studies investigating the major effects or biological activities of quercetin and the main targeted signaling pathways using in vitro and in vivo models are illustrated in Table 1.

## 5. Quercetin and microRNA

The significant impact of quercetin intake on human health is mediated principally by its capacity to influence miRNA expression. This interesting mechanism of action of quercetin participates to its protective effect. miRNAs have a regulatory role in critical processes, including inflammation, differentiation, proliferation, apoptosis and immune response as well as neurodegeneration. miRNAs interfere with the translation or degrading of target mRNAs. Interestingly, in neurogenerative disease, including AD, it has shown an altered regulation of miRNA [95].

Multiple genes could be modulated by one miRNA; concomitantly, numerous miRNAs could modulate the same gene. This interesting regulation of miRNAs favors the development of new point of view in the investigation of complex diseases, including AD. During different stages of AD and in various cells involved in this pathology [96] miRNAs are aberrantly expressed. Thus, miR-26a and miR125b levels are upregulated [96], while miR-138 [97], miR-132 [98], miR219 [99] and miRNA-15 are downregulated. Indeed, miR-132 [98] and miR-219 [99] directly target tau mRNA expression and represses tau synthesis; the elevation of miR-138 and miR125b induces tau phosphorylation [97] via GSK-3β, ERK1/2 and CDK5 activation, respectively. Interestingly, miRNA-132 is able to protect against tauopathies [100].

Similarly, quercetin affects multiple miRNAs potentially involved in AD including miR-125b, miR-26a, miR-132, miR-219, miR-15a, miR-146a, miR-9 [101,102,103,104], as well as novel miR-2218, novel miR-724, novel miR-645, novel miR-2117, novel miR-1795, novel miR-2502, novel miR-291, novel miR-1502, novel miR-2766, novel miR-1387, novel miR-345, novel miR-298, novel miR-521 and novel miR-97 [105].

A recent in vitro study showed that quercetin significantly protected PC-12 neuronal cells [105], which commonly used for the investigation of neurological diseases [106] from hydrogen peroxide-induced death. ROS are strictly linked to neurodegenerative processes [107] and act as regulators and effectors of miRNA and their target proteins. In response to oxidative stress, 135 miRNAs were found in neuronal cells and a reduced number of miRNAs is identified as key player in the antioxidant response triggered by quercetin [100]. Pretreatment of neuronal cells with quercetin prevented the altered expression of 14 miRNAs (novel miR-2218, novel miR-724, novel miR-645, novel miR-2117, novel miR-1795, novel miR-2502, novel miR-291, novel miR-1502, novel miR-2766, novel miR-1387, novel miR-345, novel miR-298, novel miR-521, novel miR-97) induced by oxidative stress. It is interesting to note that all these miRNAs linked to the antioxidant ability of quercetin were recently revealed. As a consequence, additional studies are required to determine their annotations in neurodegeneration and their precise function in the antioxidant process generated by quercetin [100].

In a model of diabetes-induced memory impairment, it has been observed the upregulation of the expression levels of miR-146a, miR-9, TNF-α, NF–kB, and subsequently amyloid-β protein precursor (AβPP), beta-site APP cleaving enzyme 1 (BACE1), and BCL2 associated X, apoptosis regulator (Bax) [104]. One of the key findings of this study is that quercetin treatment for 35 days normalized the expression of the genes in the hippocampus of diabetic rats and consequently modulating pathological inflammation. Therefore, this report suggests miRNAs/NF–kB-dependent anti-inflammatory mechanism for justifying the neuroprotective effects of quercetin in learning and memory.

Moreover, it has been shown that, at a low vitamin D grade, quercetin is efficient for enhancing cognitive dysfunction in APPswe/PS1dE9 transgenic mice (a well-recognized stable AD animal model) [103,108]. The authors suggest that, at the molecular level, the effect of quercetin might be linked to the reduction of Aβ plaques, tau phosphorylation, neuroinflammation, and overexpression of BDNF protein, and miRNAs such as miR-26a, miR-125b, and miR-132 [103].

In an AD mouse model with low vitamin D levels, supplementation with quercetin significantly reduced miR-26a and miR-125b, and increased miR-132 levels in hippocampus. This suggests that the amelioration of tau phosphorylation upon quercetin treatment might be due to the modulation of miR-26a, miR-125b, and miR132 expression [103]. The observed alterations of microRNAs were found in the hippocampus; this evidence indicates that quercetin treatment triggers the differential expression of miRNA in a specific brain region and can participate in AD development [103].

**Figure 2:**
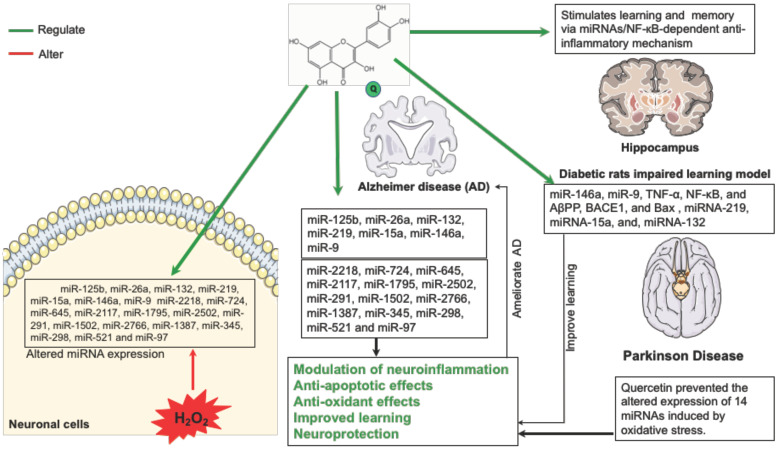
Neuroprotective and antioxidant effects of quercetin mediated by miRNA against neurological disorders. Quercetin prevents the altered expression of a number of miRNAs induced by oxidative stress in neuronal cells and PD model. Quercetin regulates miRNAs expression potentially involved in AD progress and normalized the genes and miRNAs expression in the hippocampus of diabetic rats in an impaired learning model.

Another report showed that, in aged Institute of Cancer Research (ICR) mice, cognitive impairment induced by dietary advanced glycation products (dAGEs) is improved by quercetin supplementation through enhanced tau phosphorylation and miRNAs expression in the hippocampus [109]. In the hippocampus, miRNA-219 and miRNA-15a decreased upon treatment with dAGEs; in contrast, quercetin elevates miRNA-219 and miRNA-15a, miRNA-132 levels. The authors suggested that, even if further studies are required, the improvement of tau phosphorylation upon quercetin treatment might justifying the raised levels of these miRNAs. Furthermore, they proposed that prolonged quercetin supplementation may be protective during aging, mostly with elevated levels associated with high intake of dAGEs [109]. The neuroprotective and antioxidant effects of quercetin mediated by miRNA against neurological disorders are illustrated in Figure 2.

## 6. Strategies to Improve Quercetin Effectiveness in Neurodegeneration: Synthetic and Natural Carriers 

The successful use of quercetin in the clinical treatment of neurogenerative diseases is hindered by such limitations including its insoluble nature, short half-life, low bioavailability and the difficulty to cross the BBB. Therefore, it is crucial to develop an innovative strategy targeting concomitantly the BBB-crossing ability of quercetin, as well as its bioavailability and efficacy for the treatment and the improvement of neurological outcomes.

One of most developed approaches to improve quercetin bioavailability is its encapsulation in nanocapsules, synthetic vesicles having nonimmunogenic properties, an absent toxicity, and a great biodegradability associated with the capacity to constantly release its content. Quercetin encapsulated in polylactide nanocapsules, orally administrated, efficiently reduced oxidative injury triggered by cerebral ischemia in the brain of young and old rats [110]. These nanocapsules are uptaken by the endocytic pathway, then gradually release quercetin. The mechanism by which quercetin exerts its antioxidant effect is the protection of endogenous antioxidant enzymes.

The same group showed that in a rat model of arsenic-induced toxicity, oral administration of quercetin encapsulated in nanocapsules reduced cerebral oxidative damage [111]. Moreover, formulation of encapsulated quercetin and an arsenic chelator are more efficient in the brain protection by reducing oxidative damages, improving neuronal mitochondria integrity and reducing apoptosis [112].

In a rat model of cerebral ischemia, encapsulated quercetin, orally administrated, counteracts the loss of pyramidal neurons in the hippocampus by downregulating iNOS expression and reducing the activity of caspase-3. During ischemia-reperfusion ROS sources are identified in mitochondria. Indeed, nanocapsules containing quercetin, modified in order to specifically target mitochondria, resulted in increased cerebral uptake. This formulation allows quercetin to maintain mitochondrial structure and function thus reducing ROS production and consequent apoptosis [113]. 

Another study carried out on C6 glioma cells evidenced that nanoliposomes increased quercetin bioactivity through inducing glioma necrotic cell death. Authors suggest that quercetin-nanoliposomes inhibit tumors by acting on the JAK2/STAT3 pathway and mitochondrial ROS production [114].

All these reports support the notion that the encapsulated synthetic carriers of quercetin displays improved efficacy and addressing its specific potential. Therefore, its encapsulation represents a promising toll against neurodegenerative diseases. 

Other studies have reported that in CNS, EVs, natural and heterogeneous vesicles originated from different cell types and subcellular compartments [115], act as carriers and vectors in the pathogenesis of CNS-related diseases, intercellular communication, vaccines and drug delivery. Additionally, they are functional biomarkers [109,116,117,118,119,120].

Among several elaborated approaches, the EVs emerge as innovative drug delivery system. EVs are characterized by intrinsic properties that allow to exceed limitations of synthetic nano carriers [119]. Indeed, EVs are widely investigated because of their ubiquity, and their capacity to deliver natural compounds and bioactive molecules [46]. 

In a recent study, plasma exosomes were chosen as a therapeutic cargo carrier to ensure rapid delivery of quercetin to the brain [121]. Plasma exosomes are easily and rapidly isolated from blood and they cross the BBB and induce enhanced brain migration of drug. Also, the enriched exosomes with heat shock protein 70 (HSP70) might enhance the BBB crossing by specific active targeting between exosome carrying HSP70 and endothelial TLR4 in brain. Moreover, it has been shown that plasma exosomes concur to a concomitant action on neuronal cell apoptosis reduction and cognitive decline attenuation in an okadaic acid-induced AD mouse models by downregulating hyperphosphorylation of tau protein [121].

Interestingly, quercetin has been encapsulated into plasma exosomes in order to enhance its bioavailability and capacity to target the brain. An in vivo study showed that quercetin- enriched exosomes are biocompatible and safe, as they have no overall systemic toxicity. Also, the in vivo study demonstrated that exosomes charged with quercetin modified the pharmacokinetic profiles of quercetin by increasing brain targeting and by increasing quercetin delivery and concentration into hippocampus in comparison with quercetin alone. Exosomes charged with quercetin rescued the neurodegeneration and the cognitive dysfunction of okadaic acid-induced AD mouse model by neuronal cell apoptosis reduction and by the improvement of memory and capacity in spatial learning in comparison with animals receiving quercetin alone.

From a mechanistic point of view, quercetin-enriched exosomes induced the lower expressions of cleaved caspase 9 and cleaved caspase 3, allowing the anti-apoptotic effect and blocking the assembly of insoluble neurofibrillary structures acting on the reduction of CDK5 activity and tau protein hyperphosphorylation, contributing to its neuroprotection and functional improvement [121].

This study is a clear example sustained the concept that is possible to increase the bioavailability and brain targeting of quercetin by encapsulating it into natural exosomes and that quercetin-enriched exosomes could be efficient as a potential therapeutic tool for AD therapy.

## 7. Conclusions and Future Perspectives

In summary, we reviewed the potential therapeutic roles of quercetin in neurodegenerative diseases through the suppression of oxidative stress, inflammation, microglia-mediated inflammatory responses, apoptosis and preserving BBB integrity. We also described the protective action of quercetin by influencing miRNA expression. The bioavailability of quercetin results increased with different drug delivery systems, but further studies are needed to increase its effectiveness in the brain. Given the emerging role of quercetin in counteracting the oxidative stress and neuroinflammation associated with the development of neurodegenerative disorders, the investigation of the molecular mechanisms requires further investigation.

## Figures and Tables

**Table 1:** Summary of the major effects of quercetin and their main targeted signaling pathways in vitro and in vivo models.

Biological Activities	Study Model	Major Findings	Signaling Pathways	References
Quercetin-induced apoptosis in cervical cancer cells and regulates tumorigenesis.	In vitro human cervical carcinoma HeLa cells	Quercetin exerts its suppressive, anti-proliferative and anti-migratory effect through MAPK, PI3K and WNT pathways	MAPK, PI3K and WNT pathways	[32]
Neuroprotective effect against diabetes induced nerve damage, Inducer of neuronal plasticity in the myenteric plexus	STZ-induced diabetes mellitus in rats	Quercetin treatment enhanced the bioavailability of jejunal NO bioavailability in euglycemic and diabetic rats. Quercetin prevents diabetes-induced morphological changes in the myenteric plexus of diabetic rats	Neuronal NO pathway	[36]
Anti-oxidative, anti-ER stress, neuroprotective effect against diabetic encephalopathy	db/db mouse model	Quercetin: 1. Improved learning and memory impairment 2. Alleviated impaired glucose tolerance and Insulin resistance 3. Decreased oxidative stress and protects against neuronal apoptosis in the brain of db/db mice 4. Relieved ER stress through the activation of SIRT1	SIRT1/ER stress pathway	[34]
Anti-inflammatory, anti-oxidative stress in the carotid arteries of diabetic rats	Diabetes-induced atherosclerosis rat model	Quercetin reduced hyperlipidemia, inflammatory cytokines and oxidative stress in the carotid arteries of diabetic rats on high-fat diet	AMPK/SIRT1/NF-κB signaling	[33]
Anti-apoptotic effects mediated by Nrf-2 pathway against neurotoxicity. Neuroprotective effects due to up- and/or down-regulation of cytokines	Mouse mode of neurotoxicity	Quercetin: 1. Improved behavior impairment in d-galactose-induced neurotoxicity in mice. 2. Protected hippocampus neuron from damage induced by d-galactose. 3. Activated Nrf2-ARE signaling pathway in the hippocampus of d-galactose-treated mice. 4. Ameliorates Alzheimer disease via antioxidant pathway	Nrf2, Paraoxonase-2, c-Jun N-terminal kinase (JNK), PKC, MAPK signaling cascades, and PI3K/Akt pathways.	[37,38]
Neuroprotective protective effect against the Vincristine-induced apoptosis in the sciatic nerve	Rat model of nerve injury	Quercetin reduces the ER stress caused by a vinca alkaloid antineoplastic agent (chemotherapy agent) in sciatic nerves and activates Akt, Nrf2 pathways. Quercetin may exert a protective effect against vincristine-induced peripheral neurotoxicity by suppressing NF–κB, caspase 3 and ATF-6 pathways	Akt, Nrf-2, NFκB, caspase 3, ATF-6 pathways	[90]
Neuroprotective effects due to the activation of PON2 pathway and antagonizing the oxidative-induced neuronal toxicity.	PON2 knockout mice, Mouse striatal astrocytes. CPF-induced neurotoxicity in rats	Quercetin increased PON2 expression in striatal astrocytes; Exerts neuroprotection in vitro and in vivo, JNK/AP-1 pathways. Neuroprotective effects of quercetin has been significantly reduced on cells derived from PON2 knockout mice and CPF-induced neurotoxicity in rats.	Paraoxonase 2 (PON2) pathway, JNK and AP-1 pathway	[22,91,92]

## Data Availability

Not applicable.
